# Rethinking how memories are retrieved

**DOI:** 10.7554/eLife.111126

**Published:** 2026-04-07

**Authors:** Rocco Pizzarelli, Marilena Griguoli

**Affiliations:** 1 European Brain Research Institute (EBRI) Rome Italy; 2 Institute of Molecular Biology and Pathology of the National Council of Research (IBPM CNR) Rome Italy

**Keywords:** hippocampus, memory, acetylcholine, theta oscillations, encoding, retrieval, Human

## Abstract

Patterns of neural activity called theta oscillations have a role in memory encoding but – contrary to current thinking – do not appear to have a role in memory retrieval.

**Related research article** Gedankien T, Kriegel J, Zabeh E, McDonagh D, Lega B, Jacobs J. 2026. Cholinergic blockade reveals a role for human hippocampal theta in memory encoding but not retrieval. *eLife* **14**:RP108972. doi: 10.7554/eLife.108972.

The remarkable ability of the human brain to encode and store information – and then retrieve this information as needed – relies on distinct physiological processes supported by different brain states and neuronal circuits (Hasselmo, 2006; Greene et al., 2023). A brain state is a temporary pattern of electrical activity in the brain related to cognitive functions. It arises from the simultaneous activation of large numbers of neurons, which results in waves of electrical activity – also known as oscillations – spreading across different regions of the brain (Buzsáki, 2015; Girardeau and Lopes-Dos-Santos, 2021). This process is regulated by chemical compounds called neuromodulators that are released by long-range projecting neurons and are capable of influencing thousands of neurons across multiple regions of the brain at the same time.

Released by cholinergic neurons, acetylcholine is a neuromodulator that has a key role in cognitive functions such as attention, learning and memory. The early degeneration of cholinergic neurons is also a key feature of Alzheimer’s disease (Berry and Harrison, 2023). Results based on animal studies and computational models have lead to the hypothesis that acetylcholine is essential in toggling the brain from the memory encoding state to the memory retrieval state (Hasselmo, 2006), but it has been challenging to test this hypothesis in humans. Now, in eLife, Bradley Lega (University of Texas Southwestern), Joshua Jacobs (Columbia University) and colleagues – including Tamara Gedankien as first author, Jennifer Kriegel, Erfan Zabeh and David McDonagh – report the results of a study that sheds light on the role of brain oscillations and cholinergic signalling during memory encoding and retrieval (Gedankien et al., 2026).

The subjects in the study were 12 epilepsy patients who, as part of neurosurgical treatment for epilepsy, had received intracranial electroencephalogram implants in the hippocampus and entorhinal cortex, both of which are crucial for memory encoding and retrieval (Lipton and Eichenbaum, 2008). Each of the subjects performed two experimental sessions (drug and placebo), each of which consisted of four blocks of associative recognition memory tasks.

Each block started with the memory encoding phase: pairs of words were shown to the subject, who had to asses which word was more likely to ”fit into the other”. A brief distractor task (in the form of a maths question) was next, followed by the retrieval phase. In this phase the subjects were shown two words and had to say if the pair was intact (ie, the same as a pair shown during the encoding phase), rearranged (ie, a mix of previously shown pairs), or new (ie, previously unseen words). This allowed the researchers to test for recollection, familiarity and novelty, respectively (Figure 1).

**Figure 1.**
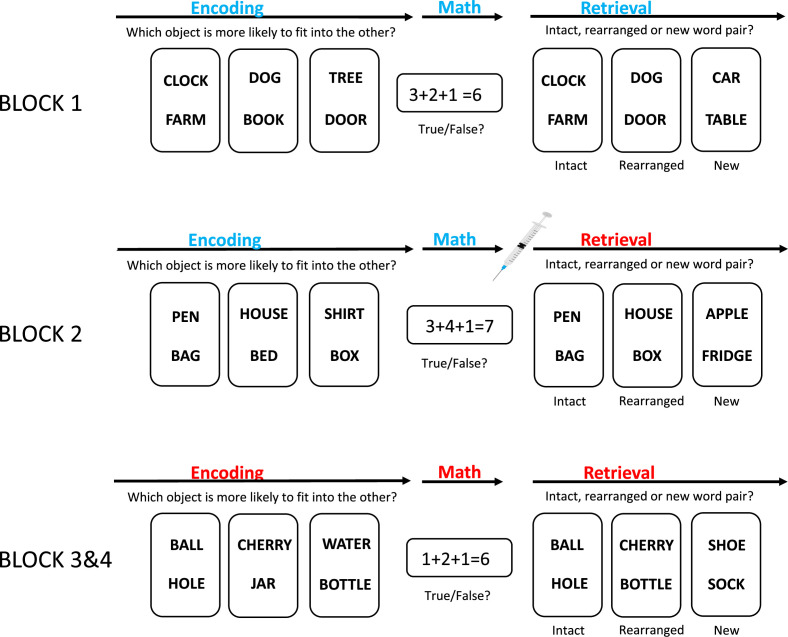
How does blocking receptors for the neuromodulator acetylcholine impact memory encoding and retrieval? Schematic showing the experimental paradigm used by Gedankien et al. During the memory encoding phase, the subject was shown a pair of words and asked to say which word was more likely to “fit into the other”. This happened 90 times. A brief mathematical distractor task was next. Then, during the retrieval phase, the subjects were shown two words and had to say if the pair was the same as a pair shown during the encoding phase (intact), a mix of previously shown pairs (rearranged), or was made of previously unseen words (new). This happened 120 times. This block was then repeated three times, with half of the subjects being injected with scopolamine, and half being injected with a placebo, during block 2 (indicated by the syringe).

During block 2 the subjects were injected with either scopolamine – a drug that blocks acetylcholine receptors – or a placebo after the distractor task. This means that half of the subjects completed the retrieval phase of block 2, and both the memory encoding and retrieval phases of blocks 3 and 4, under the influence of scopolamine, thus allowing Gedankien et al. to determine if the drug had an effect on memory encoding or retrieval. Importantly, neither the subject nor the researchers were aware if a subject had been injected with scopolamine or placebo.

Gedankien et al. found that scopolamine impaired recollection (that is, the recall of intact pairs of words) when present during the memory encoding phase, but it did not affect retrieval when administered after the memory encoding phase. This supports the idea that blocking acetylcholine receptors disrupts memory encoding but spares retrieval. Surprisingly, scopolamine also disrupted slow oscillations known as theta oscillations during memory encoding, as expected, but also during retrieval. This raises the possibility that theta oscillations do not have a role in retrieval, and are instead involved in encoding-related events that support memory updating, contextual integration and re-encoding.

The results reported by Gedankien et al. challenge our current understanding of memory function and neuronal oscillations. In the future it would be interesting to investigate the role played by regions of the brain other than the hippocampus in retrieval in the absense of acetylcholine. A better understanding of the influence of cholinergic neurons and acetylcholine on memory and theta oscillations may pave the road to therapeutic interventions for memory-related disorders.
